# Effects of aerobic and resistance exercise for 9 months on serum free light chains in type 2 diabetes

**DOI:** 10.3389/fphys.2024.1328470

**Published:** 2024-04-25

**Authors:** Youyoung Kim, John P. Campbell, Neil M. Johannsen, Timothy S. Church, Eunhan Cho, Jennifer Heaney, Guillaume Spielmann

**Affiliations:** ^1^ School of Kinesiology, Louisiana State University, Baton Rouge, LA, United States; ^2^ Department for Health, University of Bath, Bath, United Kingdom; ^3^ School of Medical and Health Sciences, Edith Cowan University, Perth, WA, Australia; ^4^ Pennington Biomedical Research Center, Baton Rouge, LA, United States; ^5^ Institute of Immunology and Immunotherapy, Birmingham, United Kingdom

**Keywords:** exercise training, free light chains, inflammation, type 2 diabetes, 9-month intervention

## Abstract

**Background and aims:** Serum polyclonal free light chains (FLCs) levels are associated with overall survival in the general population, reflecting their utility as a biomarker of underlying immune activation and inflammation. Regular exercise is known to ameliorate low-grade inflammation in chronic diseases such as type 2 diabetes; however, the effects of different exercise training modalities on FLCs in adults with type 2 diabetes is unknown. This study investigated the effects of 9-month of aerobic, resistance or combined supervised exercise on serum FLCs in 164 patients with type 2 diabetes (age 58 ± 8 years; 63% female).

**Methods:** 164 participants from the Health Benefits of Aerobic and Resistance Training in individuals with type 2 diabetes trial (HART-D) were randomly assigned to no exercise (*n* = 27), aerobic exercise alone (*n* = 41), resistance exercise alone (*n* = 49), or a combination of aerobic and resistance exercise (*n* = 47). Fasting serum samples were collected before and after completion of the intervention to quantify changes in kappa and lambda FLCs, and serum creatinine, using commercially-available ELISAs.

**Results:** At baseline, combined kappa and lambda FLCs (FLC sum; calculated as kappa + lambda FLCs) were positively correlated with high-sensitive C-reactive protein (hs-CRP) (*r* = 0.237, *p* < 0.05) and fat mass (*r* = 0.162, *p* < 0.05), and negatively associated with aerobic fitness (*r* = −0.238, *p* < 0.05). While non-exercise controls exhibited an increase in FLCs over the 9-month study, exercise training blunted this increase (Δ FLC sum control arm: 3.25 ± 5.07 mg∙L^-1^ vs. all exercise arms: -0.252 ± 6.60 mg∙L^-1^, *p* < 0.05), regardless of exercise modality.

**Conclusion:** Serum FLCs were associated with physical fitness and body composition in patients with type 2 diabetes. 9-month of exercise training prevented the accumulation of FLCs, regardless of exercise modality. Unlike hs-CRP—which did not change during the trial—serum FLCs may serve as a more sensitive biomarker of chronic low-grade inflammation in this population.

## 1 Introduction

The prevalence of type 2 diabetes has steadily increased globally over the past 30 years, and it is estimated that over 7% of the worldwide population and 15% of the United States population will have type 2 diabetes by 2030 (Rowley et al., 2017; Khan et al., 2020). Diabetes ranks in the top ten for both causes of overall mortality and burden of disease (disability-adjusted life years) (Khan et al., 2020). While the etiology of type 2 diabetes is multifaceted, it is clear that chronic activation of the immune system, and the associated chronic low-grade inflammation observed in patients with type 2 diabetes, promotes the incidence and progression of the disease (Hotamisligil, 2017). Habitual physical activity has long been advocated as a means to prevent the occurrence of type 2 diabetes (Galaviz et al., 2015), and to improve the pathophysiology of the disease such as reduced Hemoglobin A_1c_ (HbA_1c)_ (Church et al., 2010) and improved muscle metabolism (Sparks et al., 2013). However, there is still uncertainty regarding the ideal modality of exercise to improve various physiological outcomes of patients with type 2 diabetes, especially when it comes to reducing low-grade inflammation.

Biomarkers of inflammation such as circulating C-reactive protein (CRP), an indicator of innate immune activity, are known to be elevated in men and women with type 2 diabetes (Freeman et al., 2002; Nakanishi et al., 2003; Kanmani et al., 2019). However, serum CRP has been shown to exhibit profound intra- and inter-individual variability (DeGoma et al., 2012) and the mechanistic underpinnings of elevated CRP in patients with type 2 diabetes remain uncertain (Lee et al., 2009; Stanimirovic et al., 2022). To this end, novel biomarkers of inflammation such as immunoglobulin kappa and lambda free light chains (FLCs) are increasingly being explored as an alternative means of tracking chronic low-grade inflammation (Gudowska-Sawczuk and Mroczko, 2023). In healthy adults, kappa and lambda light chain isotypes are released in circulation at a steady rate of around 500 mg/day and are rapidly removed by glomerular filtration, conferring them a relatively short half-life of 2–6 h (Hutchison et al., 2014) and thus acting as a ‘real-time’ indicator of underlying immune-inflammatory activation relative to intact immunoglobulin, which has a much longer half-life. Excessive accumulation of FLCs during inflammation generates degranulation and synthesis of mast cells and releases further inflammatory mediators by activating neutrophils and other immune cells (Nakano et al., 2011; Braber et al., 2012). In this context, abnormal FLC accumulation has been reported to influence the pathogenesis of type 2 diabetes and is associated with cardiovascular diseases in this population (Bellary et al., 2014; Aberer et al., 2018; Matsumori et al., 2020). Therefore, excessive elevation of FLCs can be used to predict health and disease progression, and overall survival in general and patient populations (Dispenzieri et al., 2012).

Exercise is well-accepted to be a safe treatment for various chronic diseases, known for its ability to reduce inflammatory markers such as CRP, IL-6 and TNF- α (You and Nicklas, 2006; Hopps et al., 2011). In particular, combining aerobic with resistance exercise in patients with type 2 diabetes has been shown to be effective in reducing inflammatory cytokine concentrations by dramatically remodeling in body composition, to a greater extent than aerobic or resistance exercise in isolation (Balducci et al., 2010; Hopps et al., 2011). Furthermore, total FLCs concentrations are lower in healthy adults who report a greater level of physical activity than their sedentary age-matched counterparts, particularly those engaging in regular aerobic endurance exercise (Heaney et al., 2016). Given these findings, the practice of regular physical activity, especially combined aerobic and resistance exercise, may be a promising intervention to reduce low-grade inflammation and ameliorate FLC concentrations in patients with chronic inflammatory diseases. However, it is unknown: 1) whether exercise suppresses the accumulation of serum FLCs in patients with type 2 diabetes; 2) if exercise-associated changes in circulating FLCs are dependent on exercise modality and 3) if these changes are mediated by improvements in exercise performance and fitness or remodeling of body composition.

Therefore, the aim of this study was to investigate the effects of 9 months of aerobic, resistance or combined exercise on circulating FLCs in participants with type 2 diabetes. We hypothesized that: 1) FLCs would be related to physical fitness and hs-CRP, respectively, at trial entry, 2) FLCs would increase in non-exercising patients with type 2 diabetes over time, whereas patients enrolled in the exercise group would not exhibit increases in serum FLC concentrations, and 3) the degree by which circulating FLCs would change during the 9 months intervention would depend on exercise modality, with the greatest effect observed in the combined aerobic and resistance exercise group.

## 2 Materials and methods

### 2.1 Participants

The present study used archived serum samples from the HART-D study (Church et al., 2010). A total of 262 participants with type 2 diabetes and HbA_1c_ levels ranging from 6.5% to 11.0% were recruited for the parent study. All participants had a sedentary lifestyle, defined as less than 2 days per week of exercise, less than 60 min per week of aerobic exercise, and no resistance exercise. Of the 262 participants, a sub-cohort of 164 patients consented to be included in subsequent studies (mean ± SD: age 58 ± 8 years, female *n* = 103). Participants who did not meet the study criteria or exhibited other co-morbidities such as body mass index greater than 48.0 kg/m^2^, blood pressure greater than 160/100 mmHg, fasting triglycerides greater than 500 mg/dL, urine protein greater than 100 mg/dL, serum creatinine greater than 1.5 mg/dL, and past medical history were excluded from the parent HART-D study.

### 2.2 Study design

Participants were randomly assigned to four groups of supervised activity for 9 months: 1) a stretching-control (*n* = 27), 2) aerobic (*n* = 41), 3) resistance (*n* = 49), and 4) a combination of aerobic and resistance training groups (*n* = 47). The control group was asked to maintain present activity and was provided with optional stretching and relaxation classes once weekly.

The aerobic training group was designed for participants to perform 12 kcal/kg of body mass of walking/jogging exercise per week at 50%–80% of maximal oxygen consumption, and exercise volume was weight-adjusted every week. The resistance training was set to 3 days per week and consisted of 2 sets of 4 upper body exercises (bench press, seated row, shoulder press, and pull down), 3 sets of 3 lower body exercises (leg press, extension, and flexion), and 1 set each of abdominal crunches and back extensions. Each set consisted of 10–12 repetitions. The combined training was standardized to 10 kcal/kg per week of aerobic training and a single set of the same resistance exercises twice a week.

### 2.3 Fitness measurements

Body composition was measured by Dual-energy X-ray absorptiometry (DXA) scans using a QDR 4500A whole-body scanner (Hologic Inc., Bedford, MA). Aerobic fitness was measured using a graded exercise test on a treadmill (Trackmaster 425, Carefusion, Newton, KS).

Muscular strength measurements were performed using a Biodex System 3 dynamometer (Biodex Medical Systems, Shirley, NY). Peak torque (60°/s) and total work (300°/s) were evaluated using concentric isokinetic knee flexion and extension. Muscle quality was calculated by dividing the total amount of work performed during the 30 repetitions by the leg lean mass as determined by DXA.

### 2.4 Sample collection and analysis

Resting blood samples were collected at baseline and after the 9-month intervention using Serum Separating Tubes (BD Vacutainers). The serum samples were stored at −80 until analysis via commercially available ELISAs to characterize changes in kappa and lambda FLCs (Seralite; Abingdon Health, Oxford, United Kingdom) and hs-CRP (Abcam Cambridge, MS). Using published guidelines for hs-CRP (Haffner, 2006) and circulating FLCs (Heaney et al., 2020), 51.8% of participants had abnormal hs-CRP at baseline (greater than 3 mg∙L^-1^), and 41.5% of participants had abnormally high levels of kappa FLC (8.72–23.0 mg∙L^-1^). Creatinine and Cystatin c were measured to control for kidney function (Abcam, Cambridge, MS) according to the CKD-EPI equation (Inker et al., 2021).

### 2.5 Statistical analysis

All statistical analyzes were conducted using IBM SPSS Statistical Package Version 28.0 (IBM Inc., Armonk, NY). All data were assessed for homogeneity of variances using Levene’s test. The concentrations of hs-CRP and FLCs between baseline and 9-month values in the control were initially compared using paired t-tests to identify changes in inflammation in type 2 diabetes patients over time. The treatment effects (group and exercise modality) on hs-CRP and FLCs were determined using a two-way repeated measures ANOVA with renal function (eGFR; estimated glomerular filtration rate) as a covariate. The relationship between circulating FLCs and hs-CRP at baseline and 9-months values along with other outcome variables were initially analyzed using Pearson’s correlation. The relationship between type 2 diabetes pathophysiology, including body composition and exercise performance, along with the changes in circulating FLCs in response to the 9-month intervention were evaluated using linear regression models. The comparisons between groups, correlations, and linear regression were analyzed using the amount of change in circulating FLCs between baseline and 9-month values (Δ value; 9-month - baseline value). All results are represented as mean ± standard deviation (SD), and statistical significance was set at *p* < 0.05.

### 2.6 Ethics approval

All participants gave written informed consent before participating in the study. The study was approved by the Ethics Committee of Louisiana State University (IRB # E10505). The parent study was approved by the Ethics Committee of Pennington Biomedical Research Center and registered on clinicaltrials.gov under the Clinical trial reg. no. NCT00458133.

## 3 Results

### 3.1 Physical characteristics

The participants included in this study were a sub-sample of 164 participants [male: 37.20% (*n* = 61); female: 62.80% (*n* = 103)] from the HART-D study (Church et al., 2010). Participants’ physical characteristics are shown in Table 1. Physical characteristics between the groups were not different (*p* > 0.05).

**TABLE 1:** Baseline physical characteristics of participants.

	Total participant (*n* = 164)	Control (*n* = 27)	Aerobic (*n* = 41)	Resistance (*n* = 49)	Combination (*n* = 47)
Age (years)	57.6 ± 8.0	58.9 ± 8.8	56.7 ± 8.2	59.1 ± 8.1	55.9 ± 6.8
HbA_1c_ (%)	7.2 ± 1.1	7.5 ± 1.4	7.0 ± 0.9	7.1 ± 1.0	7.2 ± 1.1
Weight (kg)	95.6 ± 17.4	96.5 ± 21.2	93.5 ± 14.3	96.0 ± 16.1	96.6 ± 19.2
BMI (kg/m^2^)	34.1 ± 5.7	35.0 ± 6.6	33.2 ± 5.1	33.8 ± 5.4	34.7 ± 5.9
Fat Mass (kg)	36.5 ± 10.8	38.3 ± 12.4	34.6 ± 9.1	36.2 ± 10.5	37.5 ± 11.4
Lean Mass (kg)	57.2 ± 11.4	56.2 ± 11.9	56.8 ± 11.0	58.0 ± 10.9	57.4 ± 12.3
Body Fat (%)	37.6 ± 7.6	38.9 ± 7.2	36.7 ± 7.9	37.1 ± 8.1	38.1 ± 7.1
Waist Circumference (cm)	110.5 ± 12.7	109.8 ± 15.0	108.1 ± 11.2	111.3 ± 12.2	112.2 ± 13.2
VO_2peak_ (mL/kg/min)	19.7 ± 4.4	18.9 ± 3.2	20.7 ± 5.5	19.8 ± 4.6	19.2 ± 3.4
Muscle Quality (Nm/kg)	13.2 ± 3.7	12.7 ± 2.7	13.6 ± 4.2	13.1 ± 3.7	13.2 ± 3.7

Data are mean ± SD; HbA_1c_, Hemoglobin A_1c_; BMI, body mass index; VO_2peak_, peak oxygen consumption.

### 3.2 Correlation between FLCs and hs-CRP at baseline

**FIGURE 1:**
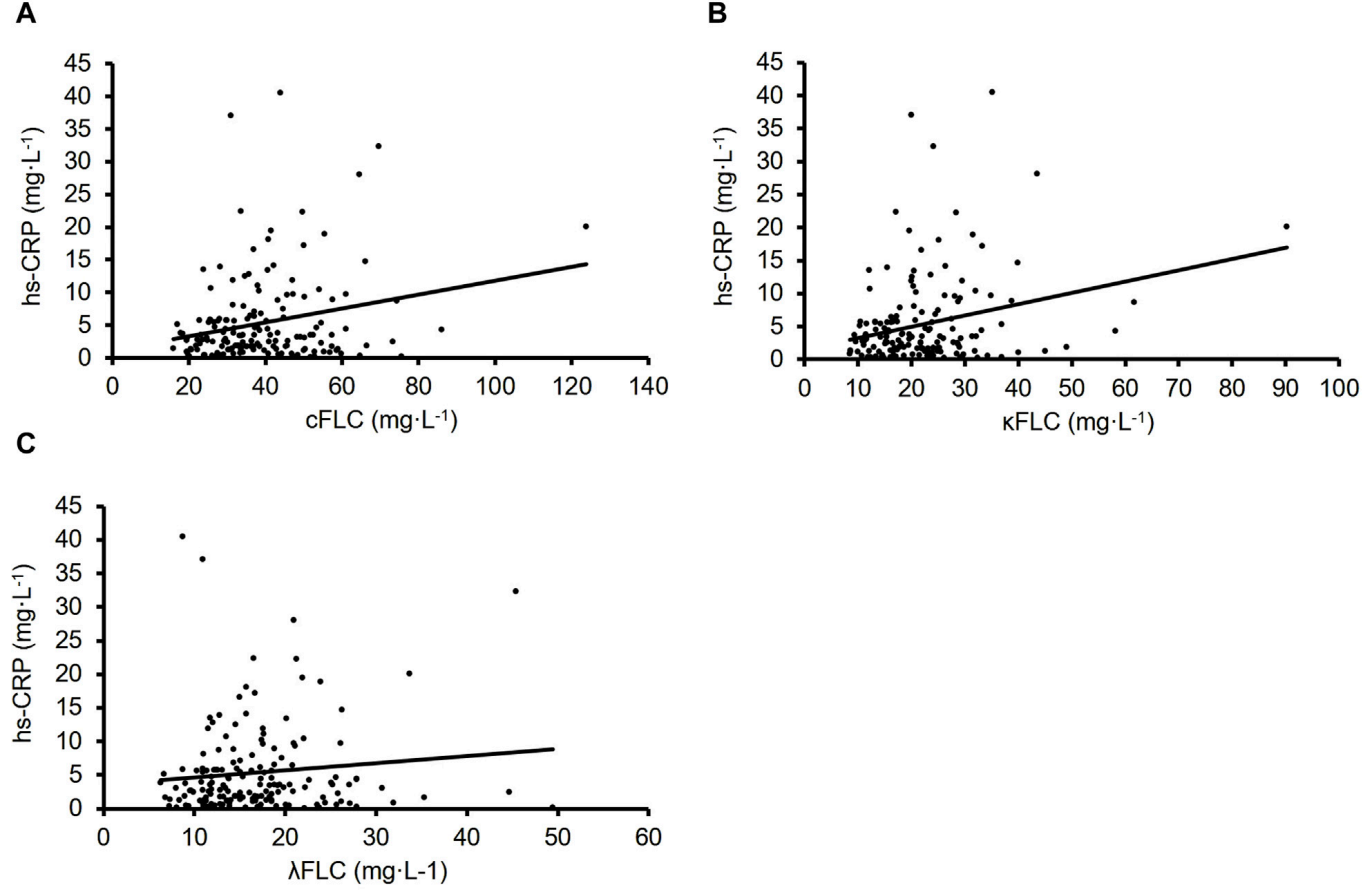
The correlation between FLCs and hs-CRP (*n* = 164). Combined FLC (*r* = 0.237, *p* < 0.05 **(A)**. Kappa FLC (*r* = 0.266, *p* < 0.05 **(B)**. Lambda FLC (*r* = 0.112, *p* > 0.05 **(C)**.

The association between serum FLCs and hs-CRP at baseline were assessed using Pearson’s correlations and shown in Figure 1. A positive correlation was observed between combined FLC (sum of kappa and lambda FLCs) and hs-CRP (*r* = 0.237, *p* < 0.05; Figure 1A). This appears to be explained by the positive correlation between kappa FLC and hs-CRP (*r* = 0.266, *p* < 0.05; Figure 1B), since no significant correlation was observed between lambda FLC and hs-CRP (*r* = 0.112, *p* > 0.05; Figure 1C).

### 3.3 Correlation between FLCs, body composition and physical fitness at baseline

Considering the impact of body composition on hs-CRP and low-grade inflammation, we investigated the correlation between FLCs levels and body composition before the start of the intervention (Table 2). Fat mass was positively correlated with combined FLC (*r* = 0.162, *p* < 0.05) and kappa FLC (*r* = 0.166, *p* < 0.05). However, circulating FLCs were not associated with any other metrics of body composition. The correlations between FLCs and exercise performance and fitness are shown in Table 3. FLCs were negatively correlated with muscle torque (*p* < 0.05) and VO_2peak_ (*p* < 0.05), independently of body composition as demonstrated by the similar association seen with muscle quality (*p* < 0.05) and lean mass VO_2peak_ (*p* < 0.05).

**TABLE 2:** Pearson’s correlation between FLCs and body composition in all participants at baseline (*n* = 164).

	Weight		Fat Mass		Lean Mass		BMI		Waist circumference	
	*r*	*p*-value	*r*	*p*-value	*r*	*p*-value	*r*	*p*-value	*r*	*p*-value
Combined FLC	0.079	0.312	0.162	0.038*	−0.028	0.723	0.098	0.210	0.010	0.901
Kappa FLC	0.079	0.312	0.166	0.034*	−0.026	0.737	0.095	0.228	0.019	0.808
Lambda FLC	0.052	0.507	0.101	0.197	−0.020	0.795	0.070	0.372	−0.008	0.923

*p < 0.05.

**TABLE 3:** Pearson’s correlation between FLCs and exercise performance and fitness in all participants at baseline (*n* = 164).

	Torque		Muscle quality		VO_2peak_		Lean Mass VO_2peak_	
	*r*	*p*-value	*r*	*p*-value	*r*	*p*-value	*r*	*p*-value
Combined FLC	-0.191	0.015*	-0.256	< 0.001*	-0.238	0.002*	-0.242	0.002*
Kappa FLC	-0.173	0.027*	-0.257	< 0.001*	-0.231	0.003*	-0.233	0.003*
Lambda FLC	-0.152	0.053	-0.168	0.031*	-0.166	0.034*	-0.172	0.028*

*p < 0.05.

### 3.4 Serum FLCs increase in inactive patients with type 2 diabetes over 9 months

**FIGURE 2:**
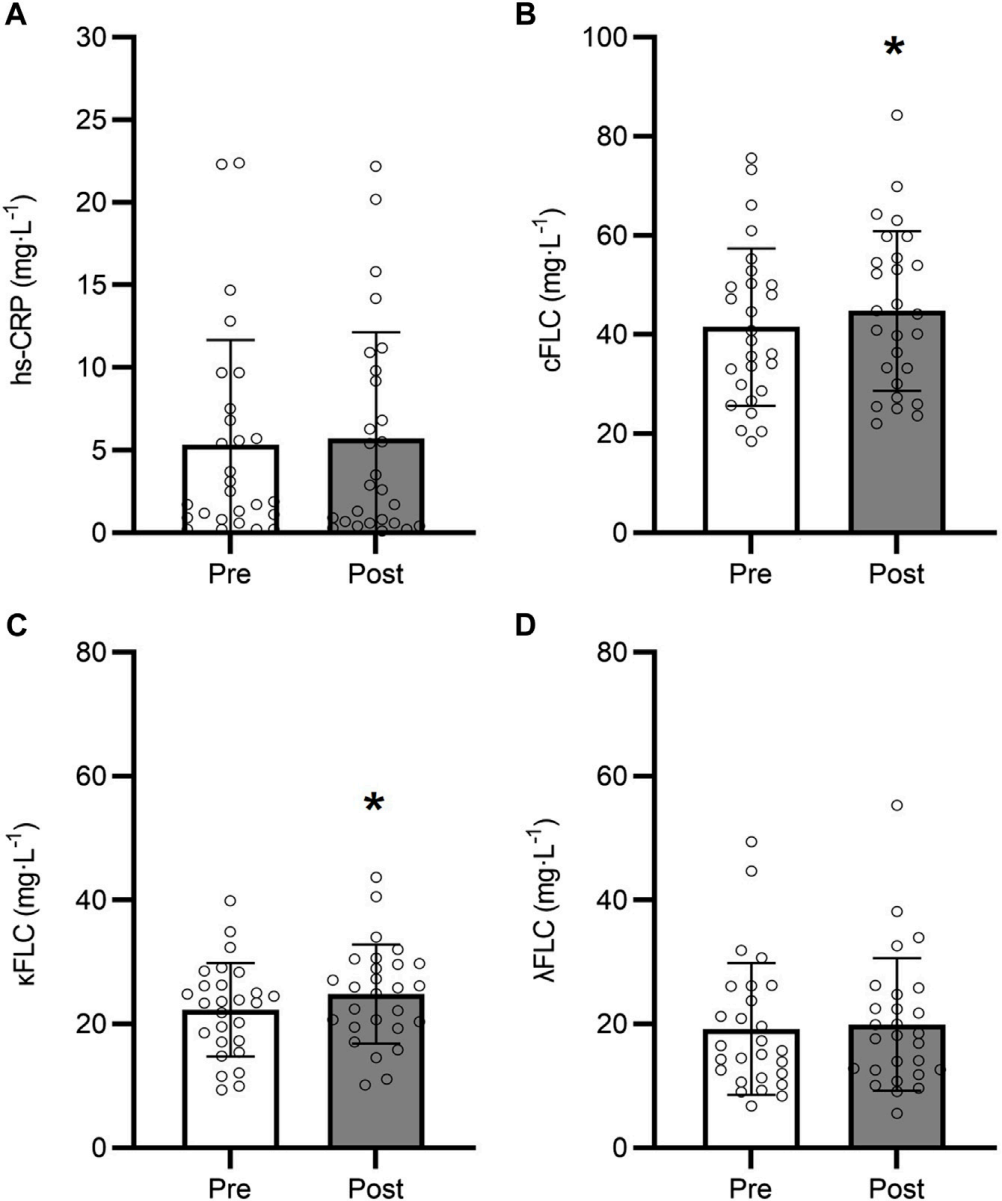
Changes in hs-CRP **(A)**, Combined FLC **(B)**, Kappa FLC **(C)**, and Lambda FLC **(D)** over the 9-month intervention in the control group (*n* = 27). *9-month different from baseline (*p* < 0.05). Data are presented as mean ± SD.

Changes in biomarkers of inflammation during the 9-month intervention in the control group are shown in Figure 2. Overall, our patient population exhibited elevated circulating hs-CRP along with kappa FLC at baseline, regardless of group. There was no difference in kappa FLC concentration between control and exercisers at baseline (*p* > 0.05), with both control and exercise participants exhibiting higher than normal levels of serum kappa FLC (frequency of participants with clinically elevated kappa FLC - control: 55.6%; exercise group: 38.7%) (Heaney et al., 2020). Interestingly, lambda FLC levels remained within the normal range for both groups. In the control group, hs-CRP was not significantly different between baseline and follow-up (*p* = 0.685; Figure 2A), however combined FLC significantly increased by 8% from 41.5 ± 15.8 mg∙L^-1^ to 44.8 ± 16.1 mg∙L^-1^ (*p* = 0.003; Figure 2B). This increase appears to be driven by kappa FLC which significantly increased by 11.7% from 22.3 ± 7.51 mg∙L^-1^ to 24.9 ± 7.99 mg∙L^-1^ (*p* < 0.001; Figure 2C), while lambda FLC levels remained unchanged at follow-up (*p* = 0.187; Figure 2D).

### 3.5 Serum FLCs and exercise training for 9 months

**FIGURE 3:**
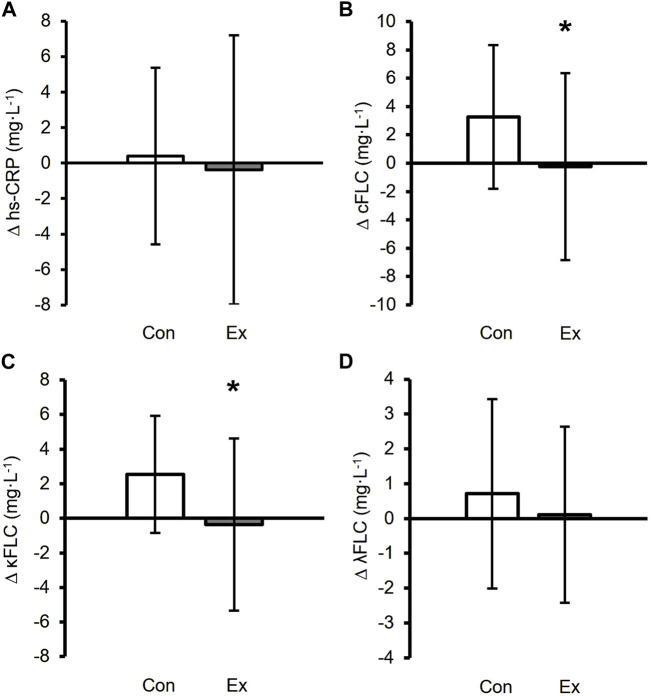
Changes in inflammatory biomarkers between the control in white (*n* = 27) and exercise groups in black (*n* = 137). ∆ values were calculated as 9-month value - baseline. ∆ hs-CRP **(A)**, ∆ Combined FLC **(B)**, ∆ Kappa FLC **(C)**, and ∆ Lambda FLC **(D)** in the control group. *Exercise group was a significant difference compared to the control group (*p* < 0.05). Data are presented as mean ± SD.

Prior to analyzing the effects of differing modalities of exercise on hs-CRP and FLCs, we characterized whether engaging in any exercise training—regardless of modality—altered circulating hs-CRP and FLCs, by comparing the inactive control groups to all the exercisers combined. The changes in FLCs and hs-CRP between the control and exercise groups in response to the 9-month intervention are shown in Figure 3. The change in hs-CRP was not significantly different between the control and exercise groups (*p* = 0.613; Figure 3A). However, the change in combined FLC in response to the 9-month exercise intervention was different between the control and exercise groups (*p* = 0.010; Figure 3B), with the change in circulating kappa FLC concentrations significantly greater in the control group than the exercisers (*p* = 0.004; Figure 3C). No differences were observed in lambda FLC between the control and exercise groups over time (*p* = 0.266; Figure 3D).

### 3.6 Exercise-induced changes in FLCs and hs-CRP are independent of exercise modality

A 2-way repeated measures ANOVA was used to identify the effects of the different exercise modalities on serum FLCs and hs-CRP in patients with Type 2 Diabetes, while controlling for change in renal function (ΔeGFR). The data are presented in Table 4. There was no difference in eGFR at baseline between the groups (*p* > 0.05), nor was there significant differences in eGFR in response to any of the interventions (*p* > 0.05). There was no main effect for time or group for hs-CRP (time: *p* = 0.820; group: *p =* 0.513), and time for lambda FLCs (time: *p* = 0.289). However, we observed a group × time interaction in serum kappa FLC (*p* = 0.039) independently of changes in kidney function. Considering the highly heterogenous nature of the patient population included in this study, we also assessed the effects of exercise modality on the changes in circulating FLCs and hs-CRP in response to 9 months of exercise training to control for baseline hs-CRP and FLCs concentrations. These are shown in Table 5. There was no significant difference in hs-CRP in response to the exercise intervention between exercise groups (*p* = 1.000). Similarly, the changes in combined (*p* = 0.865), kappa (*p* = 0.958), and lambda FLCs (*p* = 0.673) concentrations in response to the intervention were similar across the different exercise modalities.

**TABLE 4:** Mean serum FLCs and hs-CRP response in each condition. The interaction effects from the two-way repeated measures ANOVA for time (9-months) and group (exercise modality) on FLCs and hs-CRP are reported after controlling for changes in kidney function during the intervention (ΔeGFR).

	Aerobic (*n* = 41)		Resistance (*n* = 49)		Combination (*n* = 47)		Control (*n* = 27)		Interaction (*time x group*)	
	Baseline	9-month	Baseline	9-month	Baseline	9-month	Baseline	9-month	*F*	*p*
Hs-CRP (mg∙L^-1^)	5.12 ± 7.2	4.75 ± 5.8	4.40 ± 5.7	4.00 ± 5.6	6.59 ± 7.4	6.21 ± 11.0	5.33 ± 6.3	5.72 ± 6.4	0.103	0.958
Combined FLC (mg∙L^-1^)	38.73 ± 14.1	38.04 ± 15.5	36.82 ± 11.9	36.59 ± 11.9	40.04 ± 18.1	40.14 ± 15.4	41.50 ± 15.8	44.76 ± 16.1	2.338	0.076
Kappa FLC (mg∙L^-1^)	22.60 ± 11.2	22.11 ± 12.2	21.45 ± 8.7	21.03 ± 8.4	22.40 ± 12.8	22.23 ± 10.1	22.31 ± 7.5	24.85 ± 8.0	2.863	0.039*
Lambda FLC (mg∙L^-1^)	16.12 ± 5.3	15.93 ± 5.7	15.36 ± 4.6	15.56 ± 5.0	17.64 ± 7.6	17.91 ± 7.7	19.20 ± 10.6	19.91 ± 10.7	0.634	0.594

Data are presented as mean ± SD. * time × group interaction effect (*p* < 0.05).

**TABLE 5:** Changes in FLCs and hs-CRP by exercise type (*n* = 137) or control (*n* = 27).

	Aerobic (*n* = 41)	Resistance (*n* = 49)	Combination (*n* = 47)	Control (*n* = 27)
∆ hs-CRP (mg∙L^-1^)	−0.37 ± 6.0	−0.39 ± 4.1	−0.37 ± 11.0	0.39 ± 5.0
∆ Combined FLC (mg∙L^-1^)	−0.69 ± 6.0	−0.23 ± 5.3	0.10 ± 8.2	3.25 ± 5.1*
∆ Kappa FLC (mg∙L^-1^)	−0.50 ± 4.3	−0.43 ± 4.1	−0.17 ± 6.3	2.54 ± 3.4*
∆ Lambda FLC (mg∙L^-1^)	−0.19 ± 2.6	0.20 ± 2.2	0.27 ± 2.8	0.71 ± 2.7

Data are presented as mean ± SD; ∆ values were calculated as 9-month value - baseline. *9-month different from baseline (*p* < 0.05).

### 3.7 Greater reductions in FLCs are associated with improved performance and fitness in all participants

Pearson’s correlations were used to identify preliminary associations between changes in circulating FLCs levels and changes in aerobic fitness and muscle strength following the 9 months intervention (Table 6). The change in combined FLCs were negatively correlated with the changes in torque (*r* = −0.172, *p* = 0.028) and muscle quality (*r* = −0.169, *p* = 0.031) in response to the intervention. This appears to be driven by lambda FLC since the changes to lambda FLC were also negatively correlated with changes in torque (*r* = −0.197, *p* = 0.012) and muscle quality (*r* = −0.188, *p* = 0.016). However, kappa FLC was not correlated with changes in exercise performance and fitness.

**TABLE 6:** Pearson’s correlation between changes in FLCs and changes in exercise performance and fitness (*n* = 164).

	Δ Torque		Δ Muscle quality		Δ VO_2peak_		Δ Lean Mass VO_2peak_	
	*r*	*p*-value	*r*	*p*-value	*r*	*p*-value	*r*	*p*-value
Δ Combined FLC	−0.172	0.028*	−0.169	0.031*	−0.039	0.621	−0.009	0.908
Δ Kappa FLC	−0.125	0.111	−0.126	0.107	−0.060	0.448	−0.022	0.783
Δ Lambda FLC	−0.197	0.012*	−0.188	0.016*	0.015	0.850	0.018	0.817

∆values were calculated as 9-month value - baseline. *p < 0.05.

### 3.8 Greater reductions in FLCs are associated with greater improvements in performance amongst exercisers

The correlation between the change in FLCs and exercise performance over the 9-month intervention in all exercisers is shown in Table 7. Intervention-induced change in lambda FLC were negatively correlated with the changes in torque (*r* = −0.192, *p* = 0.025) and muscle quality (*r* = −0.196, *p* = 0.022) in response to the 9 months intervention. Combined and kappa FLCs were not correlated with any other exercise performance metrics.

**TABLE 7:** Pearson’s correlation between FLCs and performance based on exercise group (*n* = 137).

	Δ Torque		Δ Muscle quality		Δ VO_2peak_		Δ Lean Mass VO_2peak_	
	*r*	*p*-value	*r*	*p*-value	*r*	*p*-value	*r*	*p*-value
Δ Combined FLC	−0.133	0.121	−0.134	0.119	0.006	0.948	0.022	0.796
Δ Kappa FLC	−0.079	0.361	−0.078	0.367	0.003	0.976	0.030	0.726
Δ Lambda FLC	−0.192	0.025*	−0.196	0.022*	0.010	0.911	−0.001	0.986

∆values were calculated as 9-month value - baseline. *p < 0.05.

### 3.9 Independent variables explaining the changes in FLCs in response to the intervention

Considering the bi-directional impact of inflammation on type 2 diabetes pathophysiology, we attempted to further investigate the relationship between serum FLCs and outcomes related to the etiology of type 2 diabetes by performing multiple linear regression analysis (Table 8). The independent variables included were baseline age, sex, duration of diabetes (DB duration), kidney function using eGFR, and Δ fat mass, Δ torque, Δ muscle quality, Δ VO_2peak_, and Δ lean mass VO_2peak_. When age, sex, DB duration, eGFR and changes in body composition and performance were included, change in fat mass significantly explained the changes in kappa FLC (Model 2, β = 0.327, *R* ^2^ = 0.074, *p* = 0.022; Model 3, β = 0.343, *R* ^2^ = 0.065, *p* = 0.025; Model 4, β = 0.348, *R* ^2^ = 0.065, *p* = 0.016) between baseline and follow-up. Out of the different outcomes of performance, only the improvements in muscle quality in response to the exercise intervention significantly explained the changes in lambda FLC (Model 2, β = −0.186, *R* ^2^ = 0.059, *p* = 0.029). To identify the individual effect of the performance variables on the exercise group, multiple sub-models were run with a unique performance factor included in each (Table 9). In all models, age was a significant predictor of Δ kappa FLC concentration, with older participants exhibiting a greater reduction in kappa FLC than their younger counterparts (Model 5, β = −0.121, *R* ^2^ = 0.063, *p* = 0.050; Model 6, β = −0.120, *R* ^2^ = 0.062, *p* = 0.041; Model 7, β = −0.122, *R* ^2^ = 0.061, *p* = 0.040; Model 8, β = −0.121, *R* ^2^ = 0.061, *p* = 0.039). Conversely, sex was significantly associated with the change in lambda FLC in response to the intervention, with men exhibiting a greater decrease in lambda FLC over the 9 months intervention than women (Model 5, β = 0.951, *R* ^2^ = 0.089, *p* = 0.040; Model 7, β = 0.931, *R* ^2^ = 0.054, *p* = 0.041; Model 8, β = 0.926, *R* ^2^ = 0.054, *p* = 0.041). Finally, participants who exhibited greater improvements in muscle quality also showed the greatest reductions in lambda FLC (Model 6, β = −0.179, *R* ^2^ = 0.082, *p* = 0.050).

**TABLE 8:** Independent variables affecting the FLCs in all participants (*n* = 164).

Model 1	Δ Combined FLC			Δ Kappa FLC			Δ Lambda FLC	
	β ± SE		*p*-value	β ± SE	*p*-value		β ± SE	*p*-value
Age	−0.070 ± 0.077		0.363	−0.047 ± 0.057	0.412		−0.023 ± 0.031	0.459
Sex	1.035 ± 1.526		0.499	0.214 ± 1.145	0.852		0.822 ± 0.608	0.178
DB Duration	−0.052 ± 0.091		0.571	−0.063 ± 0.069	0.361		0.011 ± 0.036	0.763
eGFR	0.005 ± 0.019		0.775	0.008 ± 0.014	0.573		−0.003 ± 0.008	0.730
Δ Fat Mass	0.456 ± 0.277		0.102	0.394 ± 0.208	0.060		0.062 ± 0.110	0.575
Δ Torque	−0.063 ± 0.072		0.384	−0.041 ± 0.054	0.456		−0.023 ± 0.029	0.434
Δ Muscle Quality	0.179 ± 0.693		0.796	0.170 ± 0.520	0.743		0.009 ± 0.276	0.975
Δ VO_2peak_	0.476 ± 0.994		0.633	0.235 ± 0.746	0.753		0.241 ± 0.396	0.543
Δ Lean Mass VO_2peak_	−0.244 ± 0.599		0.684	−0.130 ± 0.450	0.774		−0.114 ± 0.239	0.632

Model 2	Δ Combined FLC			Δ Kappa FLC			Δ Lambda FLC	
	β ± SE		*p*-value	β ± SE	*p*-value		β ± SE	*p*-value
Age	−0.069 ± 0.073		0.344	−0.044 ± 0.055	0.418		−0.025 ± 0.029	0.394
Sex	1.222 ± 1.496		0.415	0.342 ± 1.121	0.761		0.880 ± 0.596	0.142
DB Duration	−0.063 ± 0.090		0.485	−0.070 ± 0.067	0.304		0.007 ± 0.036	0.855
eGFR	0.004 ± 0.019		0.832	0.007 ± 0.014	0.606		−0.003 ± 0.007	0.663
Δ Fat Mass	0.326 ± 0.188		0.085	0.327 ± 0.141	0.022*		0.000 ± 0.075	0.998
Δ Muscle Quality	−0.381 ± 0.212		0.074	−0.195 ± 0.159	0.222		−0.186 ± 0.084	0.029*

Model 3	Δ Combined FLC			Δ Kappa FLC			Δ Lambda FLC	
	β ± SE		*p*-value	β ± SE	*p*-value		β ± SE	*p*-value
Age	−0.085 ± 0.074		0.255	−0.053 ± 0.055	0.337		−0.032 ± 0.030	0.292
Sex	1.469 ± 1.507		0.331	0.462 ± 1.124	0.682		1.007 ± 0.603	0.097
DB Duration	−0.061 ± 0.091		0.506	−0.068 ± 0.068	0.314		0.008 ± 0.036	0.829
eGFR	0.001 ± 0.019		0.952	0.006 ± 0.014	0.680		−0.005 ± 0.008	0.538
Δ Fat Mass	0.370 ± 0.203		0.071	0.343 ± 0.152	0.025*		0.027 ± 0.081	0.744
Δ VO_2peak_	−0.006 ± 0.234		0.981	−0.020 ± 0.175	0.910		0.014 ± 0.094	0.879

Model 4	Δ Combined FLC			Δ Kappa FLC			Δ Lambda FLC	
	β ± SE		*p*-value	β ± SE	*p*-value		β ± SE	*p*-value
Age	−0.084 ± 0.074		0.254	−0.053 ± 0.055	0.339		−0.032 ± 0.030	0.285
Sex	1.472 ± 1.507		0.330	0.465 ± 1.123	0.679		1.007 ± 0.603	0.097
DB Duration	−0.060 ± 0.091		0.506	−0.068 ± 0.068	0.315		0.008 ± 0.036	0.830
eGFR	0.001 ± 0.019		0.952	0.006 ± 0.014	0.679		−0.005 ± 0.008	0.536
Δ Fat Mass	0.372 ± 0.192		0.054	0.348 ± 0.143	0.016*		0.024 ± 0.077	0.753
Δ Lean Mass VO_2peak_	0.002 ± 0.142		0.991	−0.008 ± 0.106	0.943		0.009 ± 0.057	0.872

DB, duration, duration of diabetes; eGFR, estimated glomerular filtration rate. *p < 0.05.

**TABLE 9:** Independent variable affecting the FLCs in the exercise groups only (*n* = 137).

Model 5	Δ Combined FLC		Δ Kappa FLC		Δ Lambda FLC	
	β ± SE	*p*-value	β ± SE	*p*-value	β ± SE	*p*-value
Age	−0.135 ± 0.081	0.095	−0.121 ± 0.061	0.050*	−0.015 ± 0.031	0.627
Sex	1.249 ± 1.211	0.304	0.298 ± 0.916	0.745	0.951 ± 0.459	0.040*
DB Duration	−0.019 ± 0.102	0.852	−0.028 ± 0.077	0.714	0.009 ± 0.039	0.811
eGFR	−0.006 ± 0.014	0.649	−0.001 ± 0.011	0.939	−0.006 ± 0.005	0.297
Δ Fat Mass	0.327 ± 0.317	0.305	0.285 ± 0.240	0.237	0.042 ± 0.120	0.726
Δ Torque	−0.020 ± 0.083	0.806	−0.017 ± 0.063	0.788	−0.004 ± 0.031	0.911
Δ Muscle Quality	−0.078 ± 0.790	0.921	0.086 ± 0.597	0.886	−0.164 ± 0.299	0.586
Δ VO_2peak_	0.318 ± 1.102	0.773	−0.076 ± 0.834	0.928	0.394 ± 0.418	0.348
Δ Lean Mass VO_2peak_	−0.156 ± 0.669	0.816	0.064 ± 0.506	0.899	−0.220 ± 0.254	0.387

Model 6	Δ Combined FLC		Δ Kappa FLC		Δ Lambda FLC	
	β ± SE	*p*-value	β ± SE	*p*-value	β ± SE	*p*-value
Age	−0.142 ± 0.077	0.066	−0.120 ± 0.058	0.041*	−0.022 ± 0.029	0.448
Sex	1.239 ± 1.165	0.290	0.364 ± 0.881	0.680	0.875 ± 0.443	0.051
DB Duration	−0.023 ± 0.100	0.821	−0.030 ± 0.076	0.698	0.007 ± 0.038	0.860
eGFR	−0.006 ± 0.014	0.642	−0.001 ± 0.010	0.908	−0.005 ± 0.005	0.323
Δ Fat Mass	0.258 ± 0.227	0.258	0.291 ± 0.171	0.091	−0.034 ± 0.086	0.695
Δ Muscle Quality	−0.248 ± 0.238	0.299	−0.069 ± 0.180	0.703	−0.179 ± 0.091	0.050*

Model 7	Δ Combined FLC		Δ Kappa FLC		Δ Lambda FLC	
	β ± SE	*p*-value	β ± SE	*p*-value	β ± SE	*p*-value
Age	−0.151 ± 0.078	0.054	−0.122 ± 0.059	0.040*	−0.029 ± 0.030	0.330
Sex	1.319 ± 1.172	0.262	0.389 ± 0.883	0.661	0.931 ± 0.451	0.041*
DB Duration	−0.021 ± 0.101	0.834	−0.029 ± 0.076	0.703	0.008 ± 0.039	0.840
eGFR	−0.008 ± 0.014	0.565	−0.002 ± 0.010	0.872	−0.006 ± 0.005	0.238
Δ Fat Mass	0.290 ± 0.235	0.220	0.302 ± 0.177	0.091	−0.012 ± 0.090	0.894
Δ VO_2peak_	0.020 ± 0.253	0.938	0.011 ± 0.191	0.954	0.009 ± 0.097	0.929

Model 8	Δ Combined FLC		Δ Kappa FLC		Δ Lambda FLC	
	β ± SE	*p*-value	β ± SE	*p*-value	β ± SE	*p*-value
Age	−0.152 ± 0.077	0.052	−0.121 ± 0.058	0.039*	−0.030 ± 0.030	0.311
Sex	1.315 ± 1.169	0.263	0.389 ± 0.881	0.660	0.926 ± 0.449	0.041*
DB Duration	−0.021 ± 0.101	0.833	−0.029 ± 0.076	0.703	0.008 ± 0.039	0.842
eGFR	−0.008 ± 0.014	0.565	−0.002 ± 0.010	0.869	−0.006 ± 0.005	0.243
Δ Fat Mass	0.286 ± 0.227	0.210	0.301 ± 0.171	0.081	−0.015 ± 0.087	0.865
Δ Lean Mass VO_2peak_	0.011 ± 0.154	0.945	0.016 ± 0.116	0.894	−0.005 ± 0.059	0.936

DB, duration, duration of diabetes; eGFR, estimated glomerular filtration rate. *p < 0.05.

## 4 Discussion

Physical inactivity and sedentary behaviors are known for promoting the occurrence and progression of type 2 diabetes (Hamilton et al., 2014), and systemic low-grade inflammation (Falconer et al., 2014). Numerous studies have advocated for using exercise to prevent type 2 diabetes, and to improve clinical outcomes of patients with type 2 diabetes (Sigal et al., 2006; Jorge et al., 2011). However, the impact of different exercise modalities on biomarkers of inflammation in individuals with type 2 diabetes remain mostly unclear. In this context, this study investigated the effects of a 9-month exercise intervention that included aerobic exercise alone, resistance exercise alone, or a combination of aerobic and resistance exercise on circulating free light chains (FLCs) in this patient population. We found that serum FLCs are correlated with hs-CRP, an established biomarker of inflammation in this population (Swift et al., 2012; Burmeister et al., 2014). However, while hs-CRP did not increase in the non-exercising control group over the 9-month intervention, we observed an increase in serum FLCs. While our results confirm findings from other groups that identified the relationship between type 2 diabetes and FLCs (Matsumori et al., 2020; Matsumori, 2022), this is the first study to show that all exercise training is able to prevent the increase in circulating FLCs, thus advocating for benefits of exercise, regardless of modality. Finally, this study highlighted that exercise-mediated improvements in fat mass and muscle quality in patients with type 2 diabetes may drive the amelioration of serum FLCs.

Chronic low-grade inflammation plays a preponderant role in the occurrence and progression of type 2 diabetes (Wang et al., 2013). The pro-inflammatory milieu seen in patients is believed to be at least partially linked to excess body fat (van Greevenbroek et al., 2013), and associated elevations in circulating pro-inflammatory cytokines and adipokines (Burhans et al., 2018), by dysregulating insulin production by beta cells in the pancreas (Esser et al., 2014). In addition to impairing insulin production, the chronic release of pro-inflammatory cytokine activates the JUN N-terminal kinase (JNK) and NF-κB pathways, which leads to B-cell activation by binding to the immunoglobulin kappa light chain gene enhancer (Donath and Shoelson, 2011; Matsumori, 2022). Since FLCs are byproducts of antibody production, circulating FLCs have been postulated to act as a biomarker of chronic inflammation in patients with type 2 diabetes (Matsumori et al., 2020), and a potential surrogate measure of insulin sensitivity. In this study, we found a low-level relationship between hs-CRP and FLCs, specifically kappa and combined FLCs, regardless of disease duration. This modest correlation between hs-CRP and FLCs observed in this population of patients was similar to the correlation between CRP and FLCs reported by other groups in patients with inflammatory diseases such as kidney disease (Burmeister et al., 2014; Assi et al., 2015). Notably, other studies focusing on acute inflammatory diseases, such as heart disease, have failed to report similar correlations between combined FLC and hs-CRP (Jackson et al., 2015). Considering the differences in disease etiology and nature of the low-grade inflammatory state between acute and chronic inflammatory diseases, this advocates for the greater sensitivity of serum FLCs in characterizing chronic low-grade inflammation in type 2 diabetes as opposed to hs-CRP which is also related to the inflammatory response of acute disease and acute exercise (Kasapis and Thompson, 2005; Hutchison and Landgren, 2011). Therefore, FLCs can be used as an indicator of chronic inflammation and help predict disease progression and survival risk (Dispenzieri et al., 2012).

Sedentary behavior and the associated accumulation in visceral adiposity lead to increases in cytokines and inflammatory markers such as IL-6 and CRP (Kriketos et al., 2004), especially in patients with type 2 diabetes (Amanat et al., 2020). In the current study, serum FLCs in the non-exercising control group increased by 8% and 11.7% for combined and kappa FLCs, respectively, over the 9 months intervention. Since the increased level of basal inflammation has been associated with worsening of type 2 diabetes (Barbarroja et al., 2012), this is of great clinical relevance. Interestingly, we did not observe significant changes in HbA_1c_ over the 9 months study period, nor did we find an association between HbA_1c_ changes and changes in FLCs concentration. This is in accordance with previously published report highlighting those patients with type 2 diabetes treated with IL-1β-inhibitors exhibit reductions in hs-CRP and IL-6 without changes in HbA_1c_ (Everett et al., 2018). Since elevated hs-CRP are associated with increased risks of type 2 diabetes worsening (Esser et al., 2014), reduction in chronic low-grade inflammation likely precedes changes in the disease pathophysiology. Further, our results appear to indicate that circulating FLCs could precede the increase in hs-CRP in this patient population. While future studies are required to confirm any causality, it could be hypothesized that circulating FLCs may increase at a faster rate than the deterioration of type 2 diabetes pathophysiological index. Furthermore, circulating CRP has been suggested to be more related to body composition than type 2 diabetes pathophysiology, since some studies failed to show an association between elevated CRP and type 2 diabetes severity when adjusting for adipose tissue mass (Deacon and Ebringer, 1976; Lee et al., 2009), which limits its sensitivity as a biomarker for monitoring chronic inflammation (Burmeister et al., 2014). This is supported by studies showing that circulating FLCs are a more sensitive biomarker of systemic inflammation than hs-CRP in other inflammatory diseases such as chronic kidney disease (Assi et al., 2015).

Lastly, we identified that changes in circulating FLCs in response to 9-month of exercise were mostly explained by changes in body fat mass. In this study, continuous long-term exercise reduced the fat mass by 3.6% from 36.1 ± 10.4 kg to 34.8 ± 10.1 kg, which was predictive of a decrease in combined and kappa FLCs. Higher fat mass has been reported to be closely related to insulin resistance and chronic inflammation in patients with type 2 diabetes (Annibalini et al., 2017). Furthermore, adipose tissue is known to be involved in the increase of pro-inflammatory cytokines and chemokines (Esser et al., 2014; Tsalamandris et al., 2019). Especially, excess visceral adiposity has been reported to potentially aggravate systemic inflammation by activating inflammatory pathways and immune cells including B-cell, and secreting inflammatory cytokines (Pedersen, 2017). Therefore, long-term exercise likely decreases fat mass in diabetic patients, thereby reducing FLCs and systemic inflammation by limiting B-cell activation and cytokine release from adipose tissue (Swift et al., 2012).

Finally, age and sex were associated with kappa and lambda FLCs respectively. Older participants exhibited greater diminutions in kappa FLC concentration in response to the 9-month intervention, when compared to their younger counterparts. As aforementioned, older participants lived with type 2 diabetes for longer than the younger patients in our cohort, and aging can induce FLCs accumulation regardless of disease status, so exercise would lead to greater improvements in this population. Regarding the gender effects described in this study, men exhibited greater reductions in circulating FLCs in response to the exercise intervention than women, regardless of exercise modality. While this study was not designed to investigate sex differences in FLCs concentrations before and after a 9-month exercise intervention, it can be postulated that this difference could be due to the slightly greater, albeit non-significant increase in muscle mass observed in men than in women.

The current study is not without limitations. Indeed, the analysis utilized archived serum samples from a large-scale clinical trial (HART-D study) designed to include heterogenous participants with various type 2 diabetes pathophysiology, duration of type 2 diabetes, medication types, and intake duration. These factors could have affected the chronic low-grade inflammation and exercise intervention effectiveness described in the present study (Boulé et al., 2011; Donath, 2014). Future studies should attempt to control for this variability to better understand the effects of exercise on soluble immunity and inflammation in patients with type 2 diabetes.

In conclusion, the results of this study showed that FLCs were associated with hs-CRP in patients with type 2 diabetes and may be sensitive at detecting lifestyle changes. Indeed, long-term sustained exercise dampened the accumulation of FLCs, regardless of modality—whereas hs-CRP did not change. These beneficial effects of exercise on FLCs appear to be mostly mediated by changes in body composition. The management of FLCs based on continuous exercise is expected to be effective in preventing or reducing the progression of type 2 diabetes and associated chronic low-grade inflammation.

## Data Availability

The raw data supporting the conclusion of this article will be made available by the authors, without undue reservation.
